# Higher levels of psychiatric symptomatology reported by health professionals working in medical settings in Greece

**DOI:** 10.1186/1744-859X-10-28

**Published:** 2011-10-19

**Authors:** Ioanna V Papathanasiou, Dimitrios Damigos, Venetsanos Mavreas

**Affiliations:** 1 Department of Psychiatry, School of Medicine, University of Ioannina, Ioannina, Greece; 2 Department of Nursing, Technological Educational Institute of Larissa, Larissa, Greece

## Abstract

**Background:**

Psychological distress in healthcare workers may vary across different specialties. The purpose of this study was to investigate the differences in the rate of anxiety and depression between medical and mental healthcare workers.

**Methods:**

The sample was randomly selected and consisted of 229 workers from the medical health sector and 212 from the mental health sector, aged 39.8 ± 7.9 years old. Health workers from University and General Hospitals from all over Greece participated in the study. The Greek version of the Symptoms Rating Scale For Depression and Anxiety (SRSDA) was used. Statistics were processed with SPSS v. 17.0.

**Results:**

The medical health professionals showed statistically significantly higher scores in all the subscales in comparison with the mental health sector workers, independently of years serving in the department. The rates of a possible psychiatric disorder (score over cutoff points) were significantly elevated on the Beck-21, melancholy and asthenia subscales.

**Conclusions:**

Medical healthcare workers appear to suffer from psychological distress more than their colleagues in the mental sector.

## Background

The mental health state of healthcare professionals is an important issue, influencing the services they provide [1,2]. Healthcare workers are likely to suffer from occupational stress, which may lead to serious mental and physical health problems [3]. They exhibit high prevalence rates of social dysfunction, somatization, depression and anxiety symptoms [4,5]. The psychiatric domain is considered quite a stressful work environment. Mental health professionals who work in heavily stressful mental healthcare settings are frequently subject to the violent and aggressive behavior of some patients and experience anxiety about being hurt or further intimidated [6]. Burnout syndrome and depression have been found to be highly prevalent among nurses and residents in the mental health sector, especially among nursing staff [7]. However, some studies attribute stress to organizational, rather than work-related matters, in the psychiatric field [8,9]. In comparison to mental health workers, whose stress is well established, stress in medical workers is under-recognized and understudied. Some studies argue that medical staff appear more vulnerable to psychiatric disorders than staff in the psychiatric field [10,11] and there is a clearer picture of the reasons behind psychological distress in the surgical wards and emergency departments of the medical sector. Nevertheless, stress in nurses is least studied in the area of psychology and research on comparative assessment of stress among various specialties and possible interventions is insufficient [12-14]. Nurses experience emotional situations that can cause occupational stress [15,16]. Physicians are also subjected to psychological disorders. They exhibit high levels of burnout and appear to have problems in the physical and social domain when compared to other healthcare workers [17-19].

Every event is subject to a cognitive evaluation by the subject who experiences it. Internal and external resources, which include health, beliefs, responsibility, support, social skills, and material resources determine their coping strategies and stress, as Lazarus and Folkman proposed, occurs when pressure exceeds ones perceived ability to cope and when an imbalance between demands and resources is established [20]. As considerable work pressure is reported in the medical sector, the hypothesis that medical healthcare workers will report higher levels of psychological distress will be examined in the present study.

A thorough knowledge of psychological stress in healthcare workers will allow better interventions and reorganization of healthcare services, with emphasis on the sectors that are more vulnerable. The purpose of this study is to investigate and compare levels of psychological disturbances in medical and mental healthcare personnel.

## Materials and methods

The sample consisted of 229 workers from the medical health sector and 212 from the mental health sector, who were all randomly selected; their mean age was 39.8 ± 7.9 years old. Health workers from University and General Hospitals from all over Greece participated in the study. Randomly selected hospitals from the capital city of every municipality in Greece were included in the study. Mental health as well as medical sectors of the same hospital were included. A number was assigned to every mental healthcare worker in a given hospital and a random number generator was used to produce a series of numbers equal to the desired size of each subgroup. If an individual refused to participate, the next individual was selected according to the next random number. Local ethical committees approved the study protocol. Doctors, nurses and other healthcare workers (midwives, social workers) participated in the study.

Questionnaires were sent by post to hospitals in Athens and on the islands, where a research team distributed and then collected them in other areas. A total of 45% of the questionnaires were posted and 55% were delivered in person. A preceding phone call with the directors of the departments took place in order to agree on some convenient dates (working days) for the distribution and the collection of the questionnaires. The response rate was 76% (456 out of 600 questionnaires), with 73.5% of the questionnaires (441 out of 600) completed without any missing data in the psychometric section and thus suitable for final evaluation. The Greek version of the Symptoms Rating Scale for Depression and Anxiety (SRSDA) questionnaire was used to evaluate health professionals' psychological distress [21]. It is based on the Beck Depression Inventory I (BDI-I) and has been expanded to include 42 items (double the number of BDI items); apart from the original 21 BDI items, it contains several other subscales: the asthenia subscale, the melancholia inventory, the anxiety inventory, and the mania subscale.

The composition of the SRSDA subscales is as follows: (1) the 21-item Beck Depression Scale includes items 1, 8, 11, 13, 14, 17, 18, 19, 20, 21, 22, 23, 25, 26, 27, 28, 29, 31, 32, 34, 41. These are scored: a = 0, b = 1, c = 2, d = 3. (2) The 13-item Beck Depression Scale includes items 1, 8, 11, 13, 14, 19, 20, 22, 28, 29, 32, 34 and 41. These are scored a = 0, b = 1, c = 2, d = 3. (3) The 12-item melancholia subscale includes items 8, 11, 13, 17, 19, 20, 21, 22, 26, 29, 32 and 34. These are scored a = 0, b = 1, c = 2, d = 3. (4) The 12-item asthenia subscale includes items 2, 5, 9, 17, 21, 24, 25, 27, 28, 29, 32 and 38. These are scored: a = 0, b = 1, c = 2, d = 3. (5) The 14-item anxiety subscale includes items 3, 4, 5, 12, 15, 17, 21, 24, 25, 27, 33, 39, 40 and 42. These are scored: a = 0, b = 1, c = 2, d = 3. (6) The five-item mania subscale includes items which all are graded 6, 10, 16, 30, 37. These are scored a = -1, b = 0, c = 0, d = +1.

The cutoff points established for the Greek population in each subscale are as follows: BDI-21: 14, BDI-13: 7, melancholia: 8, asthenia: 9, anxiety: 10 [21]. Regardless, all these scales are supposed to be used as screening tools rather than substitutes for an in-depth interview [22]. For the English and Greek versions of the questionnaire see: http://www.biomedcentral.com/content/supplementary/1471-244X-3-21-S1.doc and http://www.biomedcentral.com/content/supplementary/1471-244X-3-21-S2.doc, respectively.

## Statistics

Descriptive statistics were initially generated for sample characteristics. Normality was checked by the Kolmogorov-Smirnov test. The results on the subscales were mixed. Some subscales exhibited marginally normal distributions (asthenia, melancholia, anxiety) while others were clearly not normally distributed (Beck-21, Beck-13, mania). As there are articles in the literature arguing that parametric tests can successfully be applied to non-normally distributed data [23,24], data was processed with parametric tests. The t test was used to compare the means of SRSDA scores between the two health sectors. The χ^2 ^test, along with Yates correction was used to compare rates of individuals exceeding cutoff points in SRSDA subscales. Cronbach's α ranged between 0.85 and 0.90 for individual SRSDA scales. More specifically: BDI-21: 0.89, BDI-13: 0.90, melancholia: 0.89, asthenia: 0.87, anxiety: 0.88 and mania: 0.10. Similar findings have been reported by Fountoulakis *et al*. [21]. As the mania scale exhibited the lowest reliability, well below 0.60, it was excluded from the study. A general linear model with years in a department used as a covariate and the sector as a fixed factor was applied for Beck-21, and the melancholy and asthenia subscales were used as dependent variables. The number of years in the profession and the number of years in the department were collinear. As the analyses for both variables were not significantly different, the number of years in the department was chosen based on the assumption that years working in a given department is more predictive of an individual's present distress levels than the individual's total years of working (that is, current distress levels are more related to an individual's current work environment than to accumulated work stress or burnout, or the experience of working in the field in general). Statistics were processed with SPSS v. 17.0 (SPSS, Chicago, IL, USA). Statistical significance was set at *P *= 0.05.

## Results

Demographic and job features are presented in Table 1. There was no statistical difference between the two sectors regarding the participants' age (mean value 40 and 39 years for the medical and the mental health sector, respectively). The percentage of men was 25.5% (54 men) in the mental health sector and 21.5% (49 men) in the medical sector. In all 80% of the health workers in the medical sector were nurses, 11% doctors, 2.1% midwives and the remaining 6.9% consisted of various health professions. In the mental health sector nurses represented 63% of the sample, doctors (neurologists, psychiatrists) represented 14%, and other health professionals constituted 23%. The statistical analysis revealed that the workers in the medical sector had been working in each of the departments more years than their colleagues in the mental sector. Medical health professionals showed statistically significant higher scores in all the subscales in comparison with the mental sector workers, with mean values being below the cutoff scores. The cutoff points are presented as an F scale in table 2. The rates of a possible psychiatric disorder (score over cutoff points) were significantly elevated on the Beck-21, melancholy and asthenia subscales with a statistically significant difference compared to the mental health sector. The highest rate for the medical sector was on the asthenia subscale (20.3%) and for the mental health sector on the anxiety scale (12.2%)(Table 2). In Tables 3, 4 and 5 general linear models adjusted for the number of years in the department are presented, showing that Beck-21, melancholy and asthenia scores were related to the sector, independently of the number of years in the department.

**Table 1:** Demographic features of the sample

Demographic features	Medical sector		Mental health sector		Control for mean/difference	
	**Mean**	**SD**	**Mean**	**SD**	**t**	***P *value**

Age	40	7.95	39	8.19	-0.435	0.664
Years in profession	18.34	7.65	9.31	7.5	*4.683*	0
Years in department	10.52	7.65	4.07	4.27	*4.04*	0
Sex	N	%		N	%	
Men	49	21.4		54	25.5	
Women	180	78.6		158	74.5	
Profession:						
Nurses	183	80		134	63	
Doctors	25	11		29	14	
Midwives	5	2.1		0	0	
Other	16	6.9		49	23	

**Table 2:** Comparative score presentation in SRSDA subscales

SRSDA subscales	Mean ± SD		*P *value	F scale	Percentage over cutoff point		*P *value
					
	Medical sector (n = 229)	Mental health sector (n = 212)			Medical sector (n = 229)	Mental health sector (n = 212)	
Beck-21	8.23 ± 6.79	6.21 ± 5.92	< 0.05	> 14	14.2	7.5	< 0.05
Beck-13	3.96 ± 4.26	2.83 ± 3.41	< 0.05	> 7	14.3	9.9	NS
Melancholia	4.91 ± 4.44	3.44 ± 3.62	< 0.05	> 8	16	7.5	< 0.05
Asthenia	6.32 ± 4.35	4.56 ± 3.98	< 0.05	> 9	20.3	10.3	< 0.05
Anxiety	6.36 ± 4.72	4.91 ± 4.87	< 0.05	> 10	17.6	12.2	NS
t Test				χ2 test			

NS, not significant; SRSDA, Symptoms Rating Scale For Depression and Anxiety.

**Table 3:** General linear model for Beck-21 subscale (dependent variable) and sector/years in the department (fixed and covariate, respectively)

**Dependent variable: Beck-21**	**B**	**t**	***P *value**	**95% CI for B**	
				
				**Lower bound**	**Upper bound**
				
Intercept	6.784	9.37	0	5.414	8.155
Sector (medical = 1)	2.412	3.438	0.001	1.033	3.791
Years in the department	-0.099	-0.987	0.324	-0.296	0.098

**Table 4:** General linear model for asthenia subscale (dependent variable) and sector/years in the department (fixed and covariate, respectively)

Dependent variable: asthenia	B	t	*P *value	95% CI for B	
				
				Lower bound	Upper bound
Intercept	4.263	10.004	0	3.425	5.1
Sector (medical = 1)	1.959	4.568	0	1.116	2.801
Years in the department	-0.041	-0.061	0.509	-0.161	0.08

**Table 5:** General linear model for melancholia subscale (dependent variable) and sector/years in the department (fixed and covariate, respectively)

Dependent variable: melancholia	B	t	*P *value	95% CI for B	
				
				Lower bound	Upper bound
Intercept	3.979	8.835	0	5.689	9.696
Sector (medical = 1)	1.857	4.098	0	0.966	2.748
Years in the department	-0.101	-1.564	0.118	-0.229	0.026

## Discussion

The data of the present study revealed that, despite the low mean values, a considerable percentage of health professionals ranging from 14% to 20% in the medical sector scored above the cutoff points. In the mental health sector the respective percentages ranged from 7.5 to 12%. This study supported our hypothesis that medical healthcare workers would report higher levels of psychological distress than mental healthcare workers. This finding is in accordance with previous studies that showed that neuroticism, trait and state anxiety and depression are more prevalent in the medical group [10]. Working on surgical wards, in particular, proved to be highly stressful [11]. Another study showed that while normal levels of stress were present in most physicians, a disproportionate number of emergency physicians reported high levels of stress and depression and thoughts of abandoning their specialty [25]. Likewise, the few studies on the psychological problems of health professionals in Greece revealed high percentages of anxiety and depression in the medical sector [26,27].

Although the majority of the participants in the present study were nurses, samples were not homogenous; other specialties were included as well and the care settings were not proportionally representative. The different composition of the groups in terms of professions raises the question of whether the observed differences between the sectors are due to requirements of the sector or to their different composition. As many specialties were included and representation was not proportional for every health unit, it was difficult to control such variables and make any conclusions about their practical implications. While in Greece there are no autonomous emergency departments (ED), doctors and nurses from the clinics serve in EDs periodically. Unfortunately, information regarding working in the ED during the time of this survey was not obtained. Moreover, many participants did not fill in some job and demographic characteristics and so in-depth linear regression analysis could not be carried out. A further study, including proportionate samples of greater numbers from all care sectors within the medical and mental health area, could reveal particularly vulnerable groups and provide the appropriate explanation for the above findings. Emphasis should be placed on nursing shortages, nursing motivation and job satisfaction. The nursing shortage in Greece has caused nurses to work long and continuous shifts, taking up high levels of workload. Subsequently, further research into stress levels and distress among nurses might be especially warranted. A great deal of paperwork and bureaucracy add extra job demands. Years of experience and coping mechanisms are factors that should be taken into account as they are related to emotional competency. Identifying the multiple dimensions of stress in various clinical settings would allow for meaningful interventions.

## Conclusions

Medical healthcare workers appear to suffer from psychological distress more than their colleagues in the mental health sector. Evidence of high distress levels in the medical sector may help draw attention to this issue and trigger the appropriate interventions. A careful study of the work environment would be helpful in determining the factors responsible for psychological disturbances in healthcare professionals in the various care settings in Greece, and would be a starting point for further changes.

## Competing interests

The authors declare that they have no competing interests.

## Authors' contributions

IVP participated in study design, data collection and drafted the manuscript. DD participated in the design of the study and helped to draft the manuscript. VM conceived the study and critically revised it.

## References

[B1] Kikuchi Y Nakaya M Ikeda M Narita K Takeda M Nishi M Effort-reward imbalance and depressive state in nurses Occup Med (Lond) 2010 60 231 233 10.1093/occmed/kqp167 19951997

[B2] Tabolli S Ianni A Renzi C Di Pietro C Puddu P Job satisfaction, burnout and stress amongst nursing staff: a survey in two hospitals in Rome G Ital Med Lav Ergon 2006 28 49 52 19031557

[B3] Marine A Ruotsalainen J Serra C Verbeek J Preventing occupational stress in healthcare workers Cochrane Database Syst Rev 2006 18 CD002892 10.1002/14651858.CD002892.pub2 17054155

[B4] Shanafelt TD Sloan JA Habermann TM The well-being of physicians Am J Med 2003 114 513 519 10.1016/S0002-9343(03)00117-7 12727590

[B5] Weinberg A Creed F Stress and psychiatric disorder in healthcare professionals and hospital staff Lancet 2000 355 533 537 10.1016/S0140-6736(99)07366-3 10683003

[B6] Currid T Experiences of stress among nurses in acute mental health settings Nurs Stand 2009 23 40 46 28076073 10.7748/ns.23.44.40.s51

[B7] Halayem-Dhouib S Zaghdoudi L Zremdini R Maalej I Ben Béchir M Labbène R Burnout among mental health professionals: a Tunisian experience Rev Epidemiol Sante Publique 2010 58 403 408 10.1016/j.respe.2010.07.001 21094003

[B8] Ryan D Quayle E Stress in psychiatric nursing: fact or fiction? Nurs Stand 1999 14 32 35 11096858 10.7748/ns1999.11.14.8.32.c2708

[B9] Pinikahana J Happell B Stress, burnout and job satisfaction in rural psychiatric nurses: a Victorian study Aust J Rural Health 2004 12 120 125 10.1111/j.1440-1854.2004.00572.x 15200523

[B10] Dudley M Langeluddecke P Tennant C Psychological dysfunction in psychiatric and general nurse trainees J Psychosom Res 1988 32 373 381 10.1016/0022-3999(88)90020-7 3236265

[B11] Yamashita K Mental health of nurses--2 years follow-up Nihon Kango Kagakkaishi 1997 17 64 68 10426077 10.5630/jans1981.17.4_64

[B12] Pryjmachuk S Richards DA Mental health nursing students differ from other nursing students: Some observations from a study on stress and coping Int J Ment Health Nurs 2007 16 390 402 10.1111/j.1447-0349.2007.00494.x 17995510

[B13] Fagin L Carson J Leary J De Villiers N Bartlett H OMalley P West M McElfatrick S Brown D Stress, coping and burnout in mental health nurses: findings from three research studies Int J Soc Psychiatry 1996 42 102 111 10.1177/002076409604200204 8811394

[B14] Dosani S Review: stress is a problem for mental health nurses but research on interventions is insufficient Evid Based Ment Health 2003 6 126 10.1136/ebmh.6.4.126 14585799

[B15] Ben-Zur H Michael K Burnout, social support, and coping at work among social workers, psychologists, and nurses: the role of challenge/control appraisals Soc Work Health Care 2007 45 63 82 10.1300/J010v45n04_04 17954449

[B16] Salomé GM Martins MF Espósito VH Feelings of nursing professionals who work in emergency units Rev Bras Enferm 2009 62 856 862 10.1590/S0034-71672009000600009 20098877

[B17] Pompili M Innamorati M Narciso V Kotzalidis GD Dominici G Talamo A Girardi P Lester D Tatarelli R Burnout, hopelessness and suicide risk in medical doctors Clin Ter 2010 161 511 514 21181078

[B18] Tzeng DS Chung WC Fan PL Lung FW Yang CY Psychological morbidity, quality of life and their correlations among military health care workers in Taiwan Ind Health 2009 47 626 634 10.2486/indhealth.47.626 19996538

[B19] Røvik JO Tyssen R Hem E Gude T Ekeberg O Moum T Vaglum P Job stress in young physicians with an emphasis on the work-home interface: a nine-year, nationwide and longitudinal study of its course and predictors Ind Health 2007 45 662 671 10.2486/indhealth.45.662 18057809

[B20] Folkman S Lazarus RS Personal control and stress and coping processes: a theoretical analysis Personal Social Psychol 1984 46 839 852 10.1037//0022-3514.46.4.839 6737195

[B21] Fountoulakis KN Iacovides A Kleanthous S Samolis S Gougoulias K St Kaprinis G Bech P The Greek translation of the symptoms rating scale for depression and anxiety: preliminary results of the validation study BMC Psychiatry 2003 10 21 10.1186/1471-244X-3-21 PMC317317 14667249

[B22] Zung WW Richards CB Short MJ Self-rating depression scale in an outpatient clinic. Further validation of the SDS Arch Gen Psychiatry 1965 13 508 515 4378854 10.1001/archpsyc.1965.01730060026004

[B23] Vickers AJ Parametric versus non-parametric statistics in the analysis of randomized trials with non-normally distributed data BMC Med Res Methodol 2005 5 35 10.1186/1471-2288-5-35 16269081 PMC1310536

[B24] Zimmerman DW Statistical significance levels of nonparametric tests biased by heterogeneous variances of treatment groups J Gen Psychol 2000 127 354 364 10.1080/00221300009598589 11109998

[B25] Gallery ME Whitley TW Klonis LK Anzinger RK Revicki DA A study of occupational stress and depression among emergency physicians Ann Emerg Med 1992 21 58 64 10.1016/S0196-0644(05)82238-3 1539889

[B26] Tselebis A Gournas G Tzitzanidou G Panagiotou A Ilias I Anxiety and depression in Greek nursing and medical personnel Psychol Rep 2006 99 93 96 17037454 10.2466/pr0.99.1.93-96

[B27] Humpel N Caputi P Exploring the relationship between work stress, years of experience and emotional competency using a sample of Australian mental health nurses J Psychiatr Ment Health Nurs 2001 8 399 403 10.1046/j.1365-2850.2001.00409.x 11882159

